# Hyperammonemia alters membrane expression of GluA1 and GluA2 subunits of AMPA receptors in hippocampus by enhancing activation of the IL-1 receptor: underlying mechanisms

**DOI:** 10.1186/s12974-018-1082-z

**Published:** 2018-02-08

**Authors:** Lucas Taoro-Gonzalez, Yaiza M. Arenas, Andrea Cabrera-Pastor, Vicente Felipo

**Affiliations:** Laboratory of Neurobiology, Centro de Investigacion Príncipe Felipe, Eduardo Primo Yufera 3, 46012 Valencia, Spain

**Keywords:** Neuroinflammation, IL-1β, IL-1 receptor, Hyperammonemia, Hepatic encephalopathy, AMPA receptors, GluA1, GluA2, Membrane expression, Neurotransmission

## Abstract

**Background:**

Hyperammonemic rats reproduce the cognitive alterations of patients with hepatic encephalopathy, including altered spatial memory, attributed to altered membrane expression of AMPA receptor subunits in hippocampus. Neuroinflammation mediates these cognitive alterations. We hypothesized that hyperammonemia-induced increase in IL-1β in hippocampus would be responsible for the altered GluA1 and GluA2 membrane expression. The aims of this work were to (1) assess if increased IL-1β levels and activation of its receptor are responsible for the changes in GluA1 and/or GluA2 membrane expression in hyperammonemia and (2) identify the mechanisms by which activation of IL-1 receptor leads to altered membrane expression of GluA1 and GluA2.

**Methods:**

We analyzed in hippocampal slices from control and hyperammonemic rat membrane expression of AMPA receptors using the BS3 cross-linker and phosphorylation of the GluA1 and GluA2 subunits using phosphor-specific antibodies. The IL-1 receptor was blocked with IL-Ra, and the signal transduction pathways involved in modulation of membrane expression of GluA1 and GluA2 were analyzed using inhibitors of key steps.

**Results:**

Hyperammonemia reduces GluA1 and increases GluA2 membrane expression and reduces phosphorylation of GluA1 at Ser831 and of GluA2 at Ser880. Hyperammonemia increases IL-1β, enhancing activation of IL-1 receptor. This leads to activation of Src. The changes in membrane expression of GluA1 and GluA2 are reversed by blocking the IL-1 receptor with IL-1Ra or by inhibiting Src with PP2.

After Src activation, the pathways for GluA2 and GluA1 diverge. Src increases phosphorylation of GluN2B at Tyr14721 and membrane expression of GluN2B in hyperammonemic rats, leading to activation of MAP kinase p38, which binds to and reduces phosphorylation at Thr560 and activity of PKCζ, resulting in reduced phosphorylation at Ser880 and enhanced membrane expression of GluA2.

Increased Src activity in hyperammonemic rats also activates PKCδ which enhances phosphorylation of GluN2B at Ser1303, reducing membrane expression of CaMKII and phosphorylation at Ser831 and membrane expression of GluA1.

**Conclusions:**

This work identifies two pathways by which neuroinflammation alters glutamatergic neurotransmission in hippocampus. The steps of the pathways identified could be targets to normalize neurotransmission in hyperammonemia and other pathologies associated with increased IL-1β by acting, for example, on p38 or PKCδ.

**Graphical abstract:**

IL-1β alters membrane expression of GluA1 and GluA2 AMPA receptor subunits by two difrerent mechanisms in the hippocampus of hyperammonemic rats.

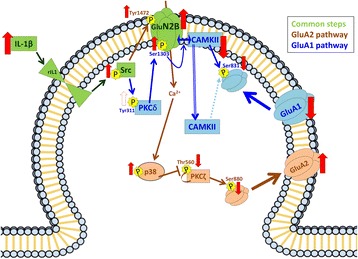

## Highlights

- Hyperammonemia reduces GluA1 and increases GluA2 membrane expression in hippocampus
- Hyperammonemia reduces phosphorylation of GluA1 in Ser831 and of GluA2 in Ser880
- These effects are mediated by IL-1β and activation of IL-1 receptor and Src kinase
- Changes in GluA2 are mediated by changes in Src, GluN2B, MAP kinase p38, and PKCζ
- Changes in GluA1 are mediated by changes in Src, GluN2B, CaMKII, and PKCδ

## Background

Patients with chronic liver diseases (cirrhosis, hepatitis...) may present hepatic encephalopathy with cognitive and motor alterations including attention deficits, mild cognitive impairment, and reduced spatial memory [1–4]. Hyperammonemia and inflammation are the main contributors to the neurological alterations in hepatic encephalopathy [5, 6]. Rats with chronic hyperammonemia similar to that present in patients with liver cirrhosis also show cognitive alterations, including impaired spatial learning and memory [7, 8]. Chronic hyperammonemia per se induces neuroinflammation, which mediates the cognitive alterations in rats [8, 9]. Neuroinflammation is also a main contributor to cognitive deficits in many chronic (e.g., cirrhosis, diabetes), mental (e.g., schizophrenia), and neurodegenerative (e.g., Alzheimer’s) diseases and in situations such as post-operative cognitive dysfunction or aging [10–15]. Neuroinflammation-induced cognitive impairment is therefore a highly and increasingly prevalent situation with serious health, social, and economic consequences. Neuroinflammation alters cognitive function by altering neurotransmission [15, 16]. The mechanisms by which neuroinflammation alters neurotransmission may share common aspects in different pathologies. Unveiling these mechanisms may provide therefore the bases to design new therapeutic approaches which could be applied in different highly prevalent pathologies.

Neuroinflammation impairs spatial learning by mechanisms which are beginning to be unveiled.

Sustained expression of IL-1β in hippocampus impairs spatial learning and memory [17, 18].

Spatial learning is mainly modulated by NMDA and AMPA receptors for glutamate in hippocampus [19, 20]. A main mechanism modulating glutamatergic neurotransmission and synaptic plasticity in hippocampus is the modulation of membrane expression of AMPA receptors, which is mainly mediated by changes in phosphorylation of the GluA1 subunit in Ser831 and Ser845 and of the GluA2 in Ser880 [21–26].

Neuroinflammation alters membrane expression of glutamate (AMPA, NMDA) and GABA receptors in hippocampus and impairs spatial learning [17, 27, 28]. Lai et al. [29] showed that exposure to IL-1β reduces phosphorylation in Ser831 and membrane expression of GluA1 in hippocampal neurons and this was prevented by IL-1Ra, an antagonist of IL-1 receptors. Machado et al. [30] also found that IL-1β reduced phosphorylation of GluA1 subunit at Ser831 and Ser845 60 min after contextual fear memory reactivation and that intra-hippocampal administration of IL-1β after memory reactivation also induced a decrease in surface expression and total expression of GluA1.

We have recently proposed that impaired spatial learning and memory in rats with hepatic encephalopathy due to portacaval shunts would be due to the increased levels of IL-1β in hippocampus [28]. We have also shown that both rats with hepatic encephalopathy and rats with hyperammonemia without liver failure show neuroinflammation, with increased levels of IL-1β and other pro-inflammatory markers, and altered membrane expression of AMPA receptor subunits GluA1 and GluA2 in hippocampus [7, 28, 31].

We hypothesize that hyperammonemia-induced increase in IL-1β in hippocampus would be responsible for the altered membrane expression of GluA1 and GluA2 subunits of AMPA receptors. The aims of this work were to (1) assess if increased IL-1β levels and activation of its receptor (IL-1R) are responsible for the changes in GluA1 and/or GluA2 membrane expression in hyperammonemia and (2) identify the mechanisms by which activation of IL-1R leads to altered membrane expression of GluA1 and GluA2.

The model of chronic hyperammonemia in rats used consisted in administering them an ammonium-containing diet as described in [32]. Membrane expression and phosphorylation of the GluA1 and GluA2 were analyzed in freshly isolated hippocampal slices from control and hyperammonemic rats. To assess the role of IL-1β in the changes in GluA1 and GluA2, we tested whether blocking the IL-1β receptor with the endogenous antagonist IL-1Ra reverses these changes. We also analyzed the intracellular pathways mediating the effects of IL-1β by assessing the effects of modulating different steps on GluA1 and GluA2 phosphorylation and membrane expression.

## Methods

### Model of chronic hyperammonemia

Male Wistar rats (120–140 g at the beginning of the diet, Charles River Laboratories, Barcelona, Spain) were made hyperammonemic by feeding them a diet containing standard diet supplemented with ammonium acetate as in [32]. In the present work, the diet contained 25% of ammonium acetate instead of 20% as in [32]. This change was made because now we house the rats in ventilated racks which were not used in [32]. These racks extract some ammonia, and the increase in ammonium acetate in the diet was necessary to reproduce the ammonia levels in blood obtained previously in un-ventilated cages using 20% ammonium acetate. The ammonium-containing diet was prepared as described in [33] (but using 25% ammonium acetate) and increased blood ammonia levels around threefold, an increase similar to that found in patients with liver cirrhosis. Rats remain hyperammonemic for long periods of time and reproduce many cognitive and motor alterations present in cirrhotic patients with hepatic encephalopathy and has allowed identifying some underlying mechanisms [7–9, 34–36]. Experiments were performed after 4–5 weeks in the ammonium diet, when the rats are 10–11 weeks old. The experiments were approved by the the Comite de Etica y Bienestar en Experimentacion Animal, Prince Felipe Research Center-Consellería de Agricultura, Generalitat Valenciana, and carried out in accordance with the European Communities Council Directive (86/609/EEC).

### Analysis of protein content and phosphorylation in hippocampal slices by western blot

In each experiment, four rats (two control and two hyperammonemic rats) were decapitated at 4–5 weeks of hyperammonemia and their brains quickly transferred to a plate where they were carefully dissected, separating the two hemispheres by cutting through the midline and extracting both hippocampi using thin spatulas. Once dissected, hippocampi were immersed immediately into ice-cold Krebs buffer (in mmol/L): NaCl 119, KCl 2.5, KH_2_PO_4_ 1, NaHCO_3_ 26.2, CaCl_2_ 2.5, and glucose 11, aerated with 95% O_2_ and 5% CO_2_ at pH 7.4. After that, hippocampi were placed longitudinally on a manual chopper and cut to obtain transverse slices (400 μm). Slices were transferred to incubation wells (15 slices in each well, from the two rats per group) without any distinction between dorsal or ventral hippocampus and incubated for 20 min at 35.5 °C in Krebs buffer for stabilization. One hundred nanogram per millileter of IL1Ra (R&D Systems cat# 1545-RA-025, Minneapolis, USA), 100 μM ifenprodil (Abcam cat# ab120111, Cambridge, MA), 20 μM SB239063 (Tocris cat# 1962, Bristol, UK), 10 μM rottlerin (Sigma cat# R5648, Darmstadt, Germany), or 10 μM PP2 (Sigma cat# P0042, Darmstadt, Germany) was added to the incubation buffer during 30 min to specifically inhibit IL-1 receptor, GluN2B, p38, PKCδ, and Src activities, respectively. After the treatments, slices were collected and homogenized by sonication for 20 s in a buffer (Tris-HCl 66 mM pH 7.4, SDS 1%, EGTA 1 mM, glycerol 10%, leupeptin 0.2 mg/mL, NaF 1 mM, Na orto-vanadate 1 mM). Samples were subjected to immunoblotting as in Felipo et al. [37], using antibodies against IL-1β (1:500, cat# AF-501-NA) from R&D Systems; Src (1:1000, cat# ab47405), Src phosphorylated at Tyr416 (1:1000, cat# ab40660), GluN2B phosphorylated at Ser1303 (1:1000, cat# ab81271), GluA2 phosphorylated at Ser880 (1:2000, cat# ab52180), and PKCζ phosphorylated at Thr560 (1:1000, cat# ab59412) from Abcam; GluN2B (1:1000, cat# 06-600), GluN2B phosphorylated at Tyr1472 (1:1000, cat# AB5403), GluA1 (1:1000, cat# 04-855), GluA1 phosphorylated at Ser831 (1:1000, cat# 04-823), and GluA2 (1:2000, cat# AB1768) from Millipore (Darmstadt, Germany); p38 (1:1000, cat# 9212) and p38 phosphorylated at Thr180/Tyr182 (1:500, cat# 9211) from Cell Signaling (Leiden, Netherlands); and PKCζ (1:2000, cat# sc-17,781) from Santa Cruz (Dallas, TX). As a control for protein loading, the same membranes used to quantify the amount of proteins were incubated with an antibody against Actin (1:5000, cat# ab6276) from Abcam. Secondary antibodies were anti-rabbit (cat# A8025), anti-goat (cat# A7650), or anti-mouse (cat# A3562) IgG, 1:4000 dilution conjugated with alkaline phosphatase from Sigma (St. Louis, MO). The images were captured using the ScanJet 5300C (Hewlett-Packard, Amsterdam, Netherlands), and band intensities quantified using the Alpha Imager 2200, version 3.1.2 (AlphaInnotech Corporation, San Francisco). Phosphorylation levels were normalized to the total amount of the respective proteins.

### Analysis of surface expression of receptors or CaMKII by cross-linking with BS3

Membrane surface expression of the GluA1 and GluA2 subunits of AMPA receptors, GluN2B subunit of NMDA receptors, and CaMKII (1:1000 primary antibody from Thermo Fisher cat# MA1-048, Waltham, MA) in whole hippocampal slices was analyzed at 4–5 weeks of hyperammonemia as described by Boudreau and Wolf [38], by cross-linking with BS3 (bis(sulfosuccinimidyl) suberate, Pierce cat# 21580, Rockford, IL). After the treatments (see above), slices were added to tubes containing ice-cold Krebs buffer with or without 2 mM BS3 and incubated for 30 min at 4 °C with gentle shacking. Cross-linking was terminated by quenching the reaction with 100 mM glycine (10 min, 4 °C). The slices were homogenized by sonication for 20 s. Samples treated or not with BS3 were analyzed by western blot as describe above. The surface expression of each subunit was calculated as the difference between the intensity of the bands without BS3 (total protein) and with BS3 (non-membrane protein) as described by Cabrera-Pastor et al. [39].

### Statistical analysis

Results are expressed as mean ± standard error. All statistical analyses were performed using the software program GraphPad Prism. Normality was assessed using the D’Agostino and Pearson Omnibus test and the Shapiro-Wilk normality tests. Differences in variances of normally distributed data were assessed using Bartlett’s test. Data were analyzed by a parametric two-way analysis of variance (ANOVA) followed by Bonferroni’s post hoc test to determine the individual and interaction effects between hyperammonemia and/or treatments on membrane expression and phosphorylation levels of proteins [40]. A confidence level of 95% was accepted as significant.

## Results

### Hyperammonemia alters membrane expression and phosphorylation of the GluA1 and GluA2 subunits of AMPA receptors in hippocampus

**Fig. 1:**
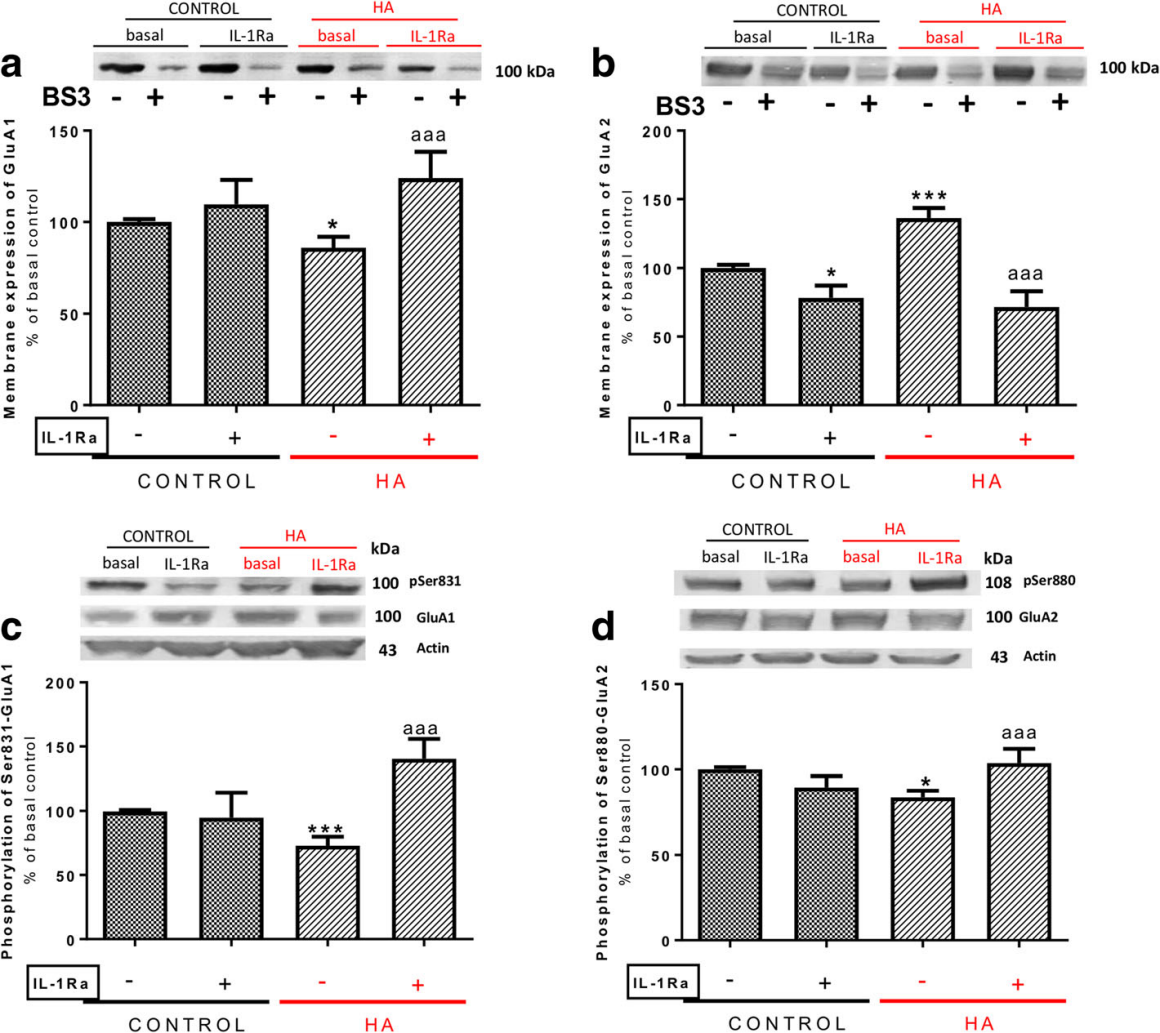
Blocking IL-1 receptor with IL-1Ra normalizes both phosphorylation and membrane expression of the GluA1 and GluA2 subunits in hyperammonemic rats. IL-1Ra, an antagonist of IL-1 receptor, was added to hippocampal slices. Membrane expression of GluA1 (**a**) and GluA2 (**b**) subunits and phosphorylation of GluA1 at Ser831 (**c**) and of GluA2 at Ser880 (**d**) were analyzed as described in the “Methods” section. Values are expressed as percentage of basal levels in control rats and are the mean ± SEM of 24, 34, 31, and 18 rats per group in **a**, **b**, **c**, and **d** respectively. Data were analyzed by two-way ANOVA. In **a**, *F* (1, 92) = 0.004596 for effect of HA, *p* = 0.9461; *F* (1, 92) = 11.77 for effect of IL1Ra, *p* = 0.0009; and *F* (1, 92) = 0.0368 for interaction, *p* = 0.0368. In **b**, *F* (1, 132) = 4.893 for effect of HA, *p* = 0.0287; *F* (1, 132) = 41.17 for effect of IL1Ra, *p* < 0.0001; and *F* (1, 132) = 9.953 for interaction, *p* = 0.0020. In **c**, *F* (1, 118) = 1.122 for effect of HA, *p* = 0.2915; *F* (1, 118) = 11.24 for effect of IL1Ra, *p* < 0.0001; and *F* (1, 118) = 15.73 for interaction, *p* = 0.0001. In **d**, *F* (1, 68) = 1.028 for effect of HA, *p* = 0.3142; *F* (1, 68) = 4.783 for effect of IL1Ra, *p* = 0.0322; and *F* (1, 68) = 22.11 for interaction, *p* < 0.0001. Values significantly different from control rats are indicated by asterisk and from hyperammonemic rats are indicated by “a”. Bonferroni post-test: ^*^p < 0.05, ^***^p < 0.001, ^aaa^*p* < 0.001

Membrane expression of the GluA1 and GluA2 subunits of AMPA receptors was altered in opposite ways in hippocampus of hyperammonemic rats. Membrane expression of GluA1 was reduced (*p* < 0.05) to 86 ± 6% in control rats (Fig. 1a), while membrane expression of GluA2 was increased (*p* < 0.001) to 136 ± 7% in control rats (Fig. 1b).

Changes in membrane expression were associated with changes in phosphorylation. Phosphorylation of GluA1 in Ser831 was reduced (*p* < 0.001) in hyperammonemic rats to 73 ± 7% in control rats (Fig. 1c), and phosphorylation of GluA2 in Ser880 was reduced (*p* < 0.05) to 84 ± 4% in control rats (Fig. 1d).

### Enhanced activation of IL-1β receptor mediates the changes in membrane expression and phosphorylation of GluA1 and GluA2 in hyperammonemic rats

As it has been shown that IL-1β modulates membrane expression of AMPA receptors, we assessed its possible contribution to the effects of hyperammonemia. In hyperammonemic rats, the content of IL-1β in hippocampus was increased to 129 ± 8% in control rats (*p* < 0.05). To assess if changes in membrane expression of GluA1 and GluA2 are due to enhanced activation of IL-1 receptor, we tested if blocking this receptor with the endogenous antagonist IL-1Ra reverses these changes. Perfusion of IL-1Ra increased (*p* < 0.001) membrane expression of GluA1 in hippocampal slices of hyperammonemic rats, reaching 124 ± 7% in control rats (Fig. 1a), in parallel with an increase (*p* < 0.001) of phosphorylation of GluA1 in Ser831 to 140 ± 16% in control rats (Fig. 1c).

IL-1Ra reduced (*p* < 0.001) membrane expression of GluA2 to the same levels in control rats (Fig. 1b) in parallel with a normalization of phosphorylation of GluA2 at Ser880 (Fig. 1d).

These data show that blocking IL-1 receptor with IL-1Ra reverses the changes induced by hyperammonemia on GluA1 and GluA2 phosphorylation and membrane expression, suggesting that enhanced activation of IL-1 receptor is responsible for these alterations in hippocampus of hyperammonemic rats.

### Activation of Src kinase mediates the effects of IL-1β on GluA1 and GluA2

**Fig. 2:**
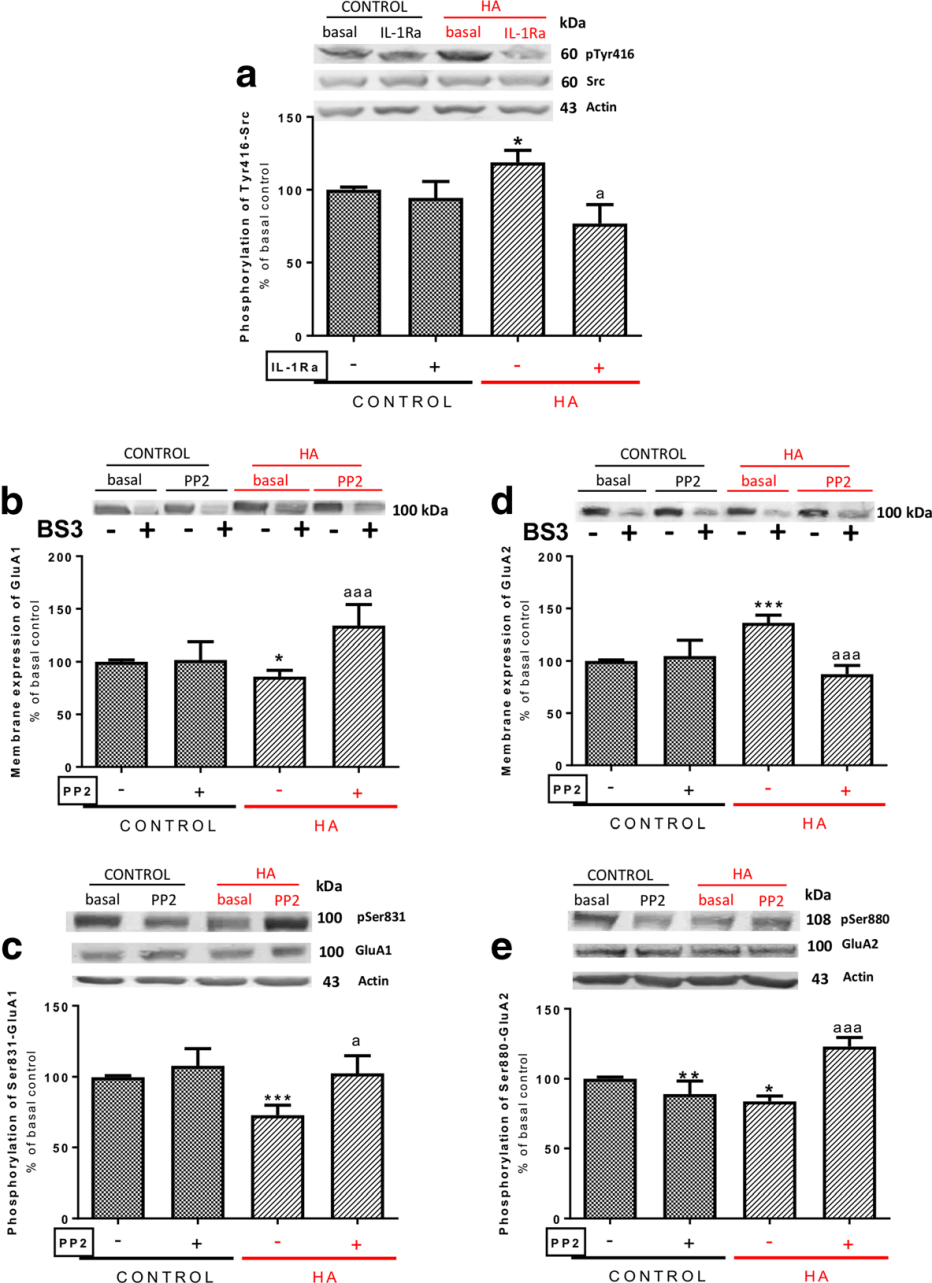
IL-1 receptor-mediated activation of Src leads to the alterations in membrane expression and phosphorylation of GluA1 and GluA2 subunit in hyperammonemic rats. IL-1Ra or PP2, an inhibitor of Src kinase, were added to hippocampal slices. Phosphorylation of Src at Tyr416 (**a**), of GluA1 at Ser831 (**c**), of GluA2 at Ser880 (**e**), and membrane expression of GluA1 (**b**) and GluA2 (**d**) were analyzed as described in the “Methods” section. Values are expressed as percentage of basal levels in control rats and are the mean ± SEM of 24, 22, 33, 32, and 18 rats per group in **a**, **b**, **c**, **d**, and **e** respectively. Data were analyzed by two-way ANOVA. In **a**, *F* (1, 92) = 0.005730 for effect of HA, *p* = 0.9398; *F* (1, 92) = 5.434 for effect of IL1Ra, *p* = 0.0219; and *F* (1, 92) = 3.058 for interaction, *p* = 0.0837. In **b**, *F* (1, 83) = 1.361 for effect of HA, *p* = 0.2466; *F* (1, 83) = 9.148 for effect of PP2, *p* = 0.0033; and *F* (1, 83) = 8.418 for interaction, *p* = 0.0048. In **c**, *F* (1, 127) = 4.230 for effect of HA, *p* = 0.0418; *F* (1, 127) = 5.912 for effect of PP2, *p* = 0.0164; and *F* (1, 127) = 2.146 for interaction, *p* = 0.1454. In **d**, *F* (1, 123) = 1.545 for effect of HA, *p* = 0.2162; *F* (1, 123) = 8.038 for effect of PP2, *p* = 0.0054; and *F* (1, 123) = 11.17 for interaction, *p* = 0.0011. In **e**, *F* (1, 68) = 7.756 for effect of HA, *p* = 0.0069; *F* (1, 68) = 7.864 for effect of PP2, *p* = 0.0066; and *F* (1, 68) = 44.50 for interaction, *p* < 0.0001. Values significantly different from control rats are indicated by asterisk and from hyperammonemic rats are indicated by “a”. Bonferroni post-test: ^*^p < 0.05, ^**^p < 0.01, ^***^p < 0.001, ^a^*p* < 0.05, ^aaa^*p* < 0.001

We then looked for the intracellular pathways mediating the changes in membrane expression induced by enhanced activation of IL-1Ra. As activation of IL-1 receptor leads often to increased phosphorylation and activity of Src kinase [41], we tested whether it is increased in hyperammonemic rats. Phosphorylation of Src at Tyr416 is increased (*p* < 0.05) in hyperammonemic rats to 118 ± 8% in control rats, and blocking IL-1 receptor with IL-1Ra normalizes it, returning to 77 ± 13% in control rats (Fig. 2a).

To assess if enhanced activation of Src mediates the changes in membrane expression of GluA1 and GluA2 in hyperammonemic rats, we tested if inhibiting Src with PP2 reverses these changes.

Perfusion of PP2 increased (*p* < 0.001) membrane expression of GluA1 in hippocampal slices of hyperammonemic rats, reaching 134 ± 20% in control rats (Fig. 2b), in parallel with an increase (*p* < 0.05) of phosphorylation of GluA1 in Ser831 to 103 ± 13% in control rats (Fig. 2c).

PP2 reduced (*p* < 0.001) membrane expression of GluA2 to the same levels in control rats (Fig. 2d) in parallel with an increase of phosphorylation of GluA2 at Ser880 (Fig. 2e).

These data suggest that enhanced activation of Src contributes to altered phosphorylation and membrane expression of GluA1 and GluA2 in hippocampus of hyperammonemic rats.

### GluN2B, p38, and PKCζ mediate the effects of IL-1β and Src on GluA2 but not on GluA1

On the bases of the knowledge in the literature (see the “Discussion” section), we hypothesized that Src would be altering GluA1 and GluA2 membrane expression by modulating the GluN2B subunit of NMDA receptors. Src phosphorylates GluN2B at Tyr1472 and this increases its membrane expression [42].

**Fig. 3:**
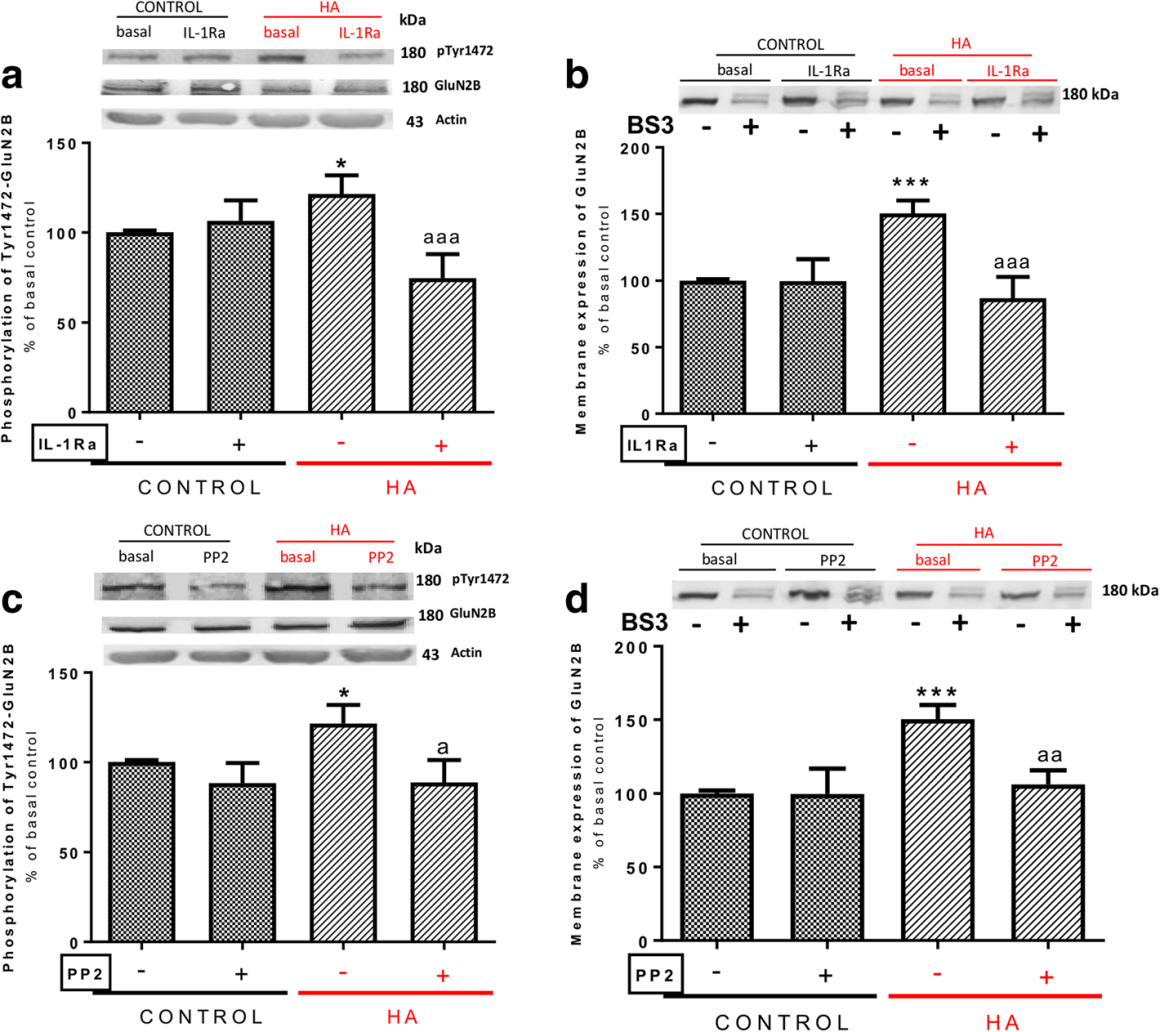
IL-1 receptor and Src activation lead to increased phosphorylation at Tyr1472 and membrane expression of the GluN2B subunit in hyperammonemic rats. IL-1Ra or PP2, an inhibitor of Src kinase, were added to hippocampal slices. Phosphorylation of GluN2B at Tyr1472 (**a**, **c**) and membrane expression of GluN2B (**b**, **d**) were analyzed as described in the “Methods” section. Values are expressed as percentage of basal levels in control rats and are the mean ± SEM of 27, 37, 28, and 37 rats per group in **a**, **b**, **c**, and **d** respectively. Data were analyzed by two-way ANOVA. In **a**, *F* (1, 104) = 0.3170 for effect of HA, *p* = 0.5746; *F* (1, 104) = 5.592 for effect of IL-1Ra, *p* = 0.0199; and *F* (1, 104) = 9.208 for interaction, *p* = 0.0030. In **b**, *F* (1, 143) = 3.642 for effect of HA, *p* = 0.0583; *F* (1, 143) = 10.33 for effect of IL-1Ra, *p* = 0.0016; and *F* (1, 143) = 9.704 for interaction, *p* = 0.0022. In **c**, *F* (1, 106) = 1.727 for effect of HA, *p* = 0.1917; *F* (1, 106) = 6.991 for effect of PP2, *p* = 0.0094; and *F* (1, 106) = 1.457 for interaction, *p* = 0.2302. In **d**, *F* (1, 143) = 8.814 for effect of HA, *p* = 0.0035; *F* (1, 143) = 5.432 for effect of PP2, *p* = 0.0212; and *F* (1, 143) = 4.963 for interaction, *p* = 0.0275. Values significantly different from control rats are indicated by asterisk and from hyperammonemic rats are indicated by “a”. Bonferroni post-test: ^*^p < 0.05, ^***^p < 0.001, ^a^*p* < 0.05, ^aa^*p* < 0.01, ^aaa^*p* < 0.001

As shown in Fig. 3, hyperammonemia increases phosphorylation (*p* < 0.05) of GluN2B at Tyr1472 to 122 ± 10% in control rats (Fig. 3a) and membrane expression (*p* < 0.001) of GluN2B to 151 ± 10% in control rats (Fig. 3b).

Blocking IL-1 receptor with IL-1Ra normalized both phosphorylation (Fig. 3a) and membrane expression (Fig. 3b) of GluN2B in hyperammonemic rats.

Similar effects were obtained in experiments using the Src inhibitor PP2, which normalized both phosphorylation (Fig. 3c) and membrane expression (Fig. 3d) of GluN2B in hyperammonemic rats.

These data support that enhanced activation of IL-1 receptor in hyperammonemia leads to increased activity of Src which phosphorylates and enhances membrane expression of GluN2B.

**Fig. 4:**
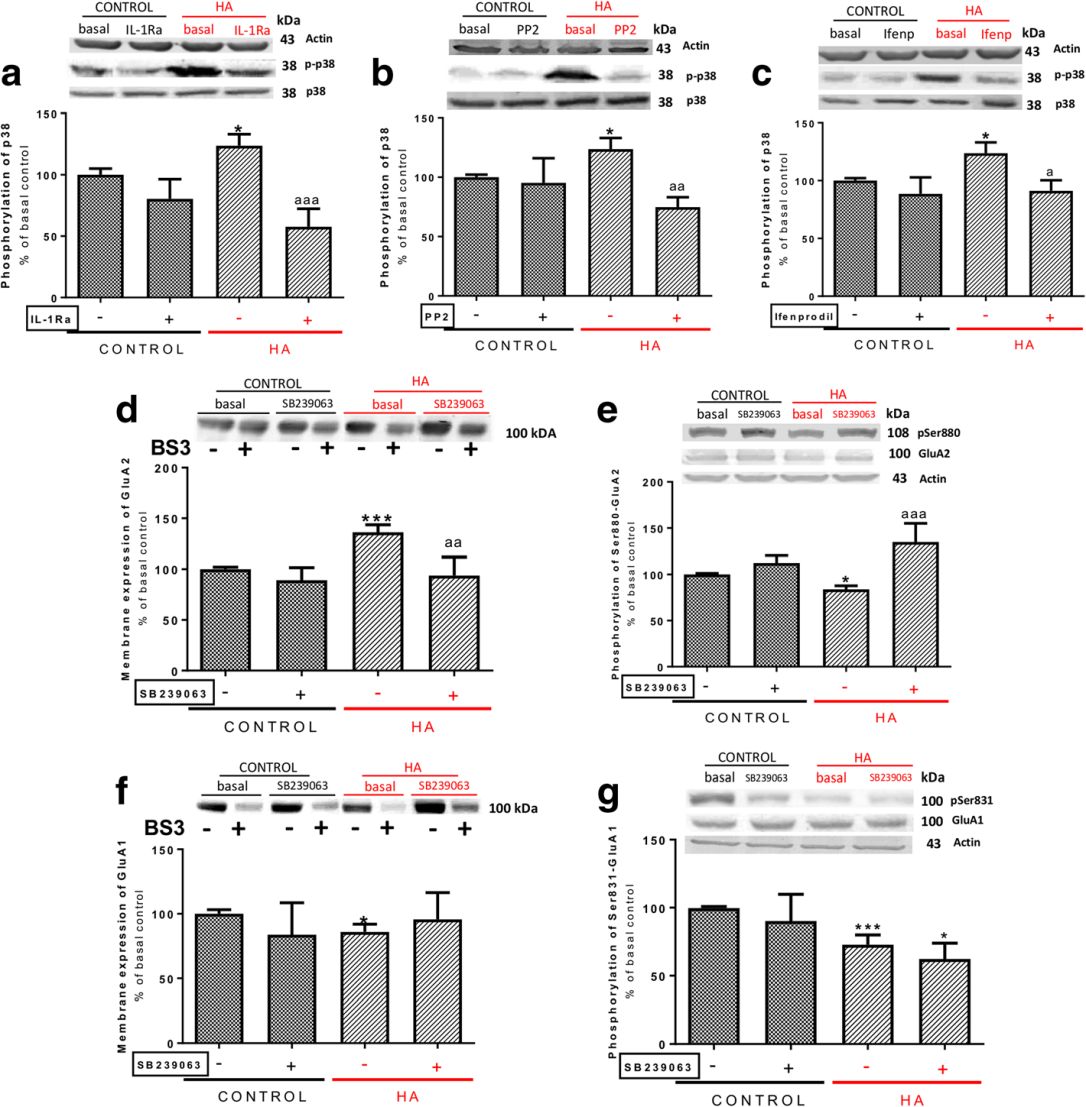
IL-1 receptor, Src, and GluN2B-mediated activation of p38 leads to the alterations in membrane expression and phosphorylation of the GluA2 but not of the GluA1 subunit in hyperammonemic rats. IL-1Ra, PP2, SB239063, an inhibitor of p38 MAP-kinase, or ifenprodil, an antagonist of GluN2B-containing NMDA receptors, were added to hippocampal slices. Phosphorylation of p38 (**a**, **b**, **c**), GluA1 at Ser831 (**g**), and GluA2 at Ser880 (**e**) and membrane expression of GluA1 (**f**) and GluA2 (**d**) subunits were analyzed as described in the “Methods” section. Values are expressed as percentage of basal levels in control rats and are the mean ± SEM of 18, 16, 20, 30, 17, 22, and 29 rats per group in **a**, **b**, **c**, **d**, **e**, **f**, and **g** respectively. Data were analyzed by two-way ANOVA. In **a**, *F* (1, 66) = 0.004793 for effect of HA, *p* = 0.9450; *F* (1, 66) = 23.42 for effect of IL-1Ra, *p* < 0.0001; and *F* (1, 66) = 6.695 for interaction, *p* = 0.0119. In **b**, *F* (1, 59) = 0.02867 for effect of HA, *p* = 0.8661; *F* (1, 59) = 6.959 for effect of PP2, *p* = 0.0106; and *F* (1, 59) = 4.611 for interaction, *p* = 0.0359. In **c**, *F* (1, 76) = 2.894 for effect of HA, *p* = 0.0930; *F* (1, 76) = 7.961 for effect of ifenprodil, *p* = 0.0061; and *F* (1, 76) = 1.682 for interaction, *p* = 0.1985. In **d**, *F* (1, 116) = 4.486 for effect of HA, *p* = 0.0363; *F* (1, 116) = 7.577 for effect of SB239063, *p* = 0.0069; and *F* (1, 116) = 2.616 for interaction, *p* = 0.1085. In **e**, *F* (1, 64) = 0.2959 for effect of HA, *p* = 0.5883; *F* (1, 64) = 26.34 for effect of SB239063, *p* < 0.0001; and *F* (1, 64) = 10.08 for interaction, *p* = 0.0023. In **f**, *F* (1, 83) = 0.006480 for effect of HA, *p* = 0.9360; F (1, 83) = 0.1665 for effect of SB239063, *p* = 0.6843; and *F* (1, 83) = 2.228 for interaction, *p* = 0.1393. In **g**, *F* (1, 110) = 6.864 for effect of HA, *p* = 0.0100; *F* (1, 110) = 0.9431 for effect of SB239063, *p* = 0.3336; and *F* (1, 110) = 0.001793 for interaction, *p* = 0.9663. Values significantly different from control rats are indicated by asterisk and from hyperammonemic rats are indicated by “a”. Bonferroni post-test: ^*^p < 0.05, ^***^p < 0.001, ^a^*p* < 0.05, ^aa^*p* < 0.01, ^aaa^*p* < 0.001

It has been reported that enhanced membrane expression of GluN2B leads to enhanced phosphorylation and activity of the MAP kinase p38 [43]. We therefore assessed whether phosphorylation of p38 was increased in hippocampus of hyperammonemic rats. Phosphorylation of p38 was increased (*p* < 0.05) to 124 ± 10% in control rats (Fig. 4) and was normalized by treatment with IL-1Ra (Fig. 4a), the Src inhibitor PP2 (Fig. 4b), or with ifenprodil (Fig. 4c), a selective antagonist of NMDA receptors containing the GluN2B subunit [44].

To assess if enhanced activation of p38 mediates the changes in membrane expression of GluA1 and GluA2 in hyperammonemic rats, we tested if inhibiting p38 with SB239063 reverses these changes.

Perfusion of SB239063 reduced (*p* < 0.01) membrane expression of GluA2 to the same levels in control rats (Fig. 4d) in parallel with an increase of phosphorylation of GluA2 at Ser880 (Fig. 4e). However, SB239063 did not normalized phosphorylation (Fig. 4g) or membrane expression (Fig. 4f) of the GluA1 subunit.

These data suggest that enhanced activation of p38 contributes to altered phosphorylation and membrane expression of GluA2 but not of GluA1in hippocampus of hyperammonemic rats.

The GluA2 subunit is phosphorylated at Ser880 by protein kinase C (PKC) [45]. The above data suggest that enhanced p38 activity would reduce the activity of some isoform of PKC, resulting in reduced phosphorylation of Ser880 and increased membrane expression of GluA2. It has been shown that activated p38 binds to PKCζ and this prevents auto-phosphorylation of PKCζ at Thr560, thus reducing its activity [46].

**Fig. 5:**
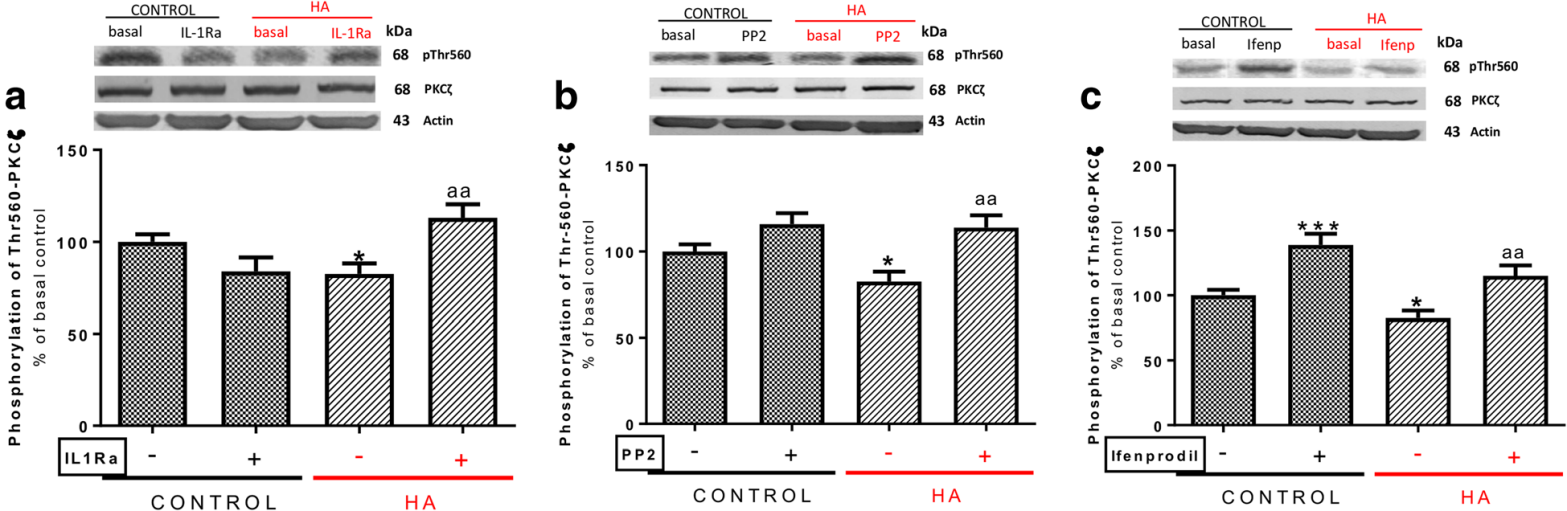
Enhanced activation of IL-1 receptor, Src, and GluN2B-containing NMDA receptors leads to reduced phosphorylation of PKCζ at Thr560 in hyperammonemic rats. IL-1Ra, PP2, or ifenprodil were added to hippocampal slices. Phosphorylation of PKCζ at Thr560 was analyzed as described in the “Methods” section. Values are expressed as percentage of basal levels in control rats and are the mean ± SEM of 15 rats per group in **a**, **b**, and **c**. Data were analyzed by two-way ANOVA. In **a**, *F* (1, 55) = 1.077 for effect of HA, *p* = 0.3039; *F* (1, 55) = 1.220 for effect of IL-1Ra, *p* = 0.2742; and *F* (1, 55) = 14.93 for interaction, *p* = 0.0003. In **b**, *F* (1, 56) = 2.647 for effect of HA, *p* = 0.1093; *F* (1, 56) = 15.57 for effect of PP2, *p* = 0.0002; and *F* (1, 56) = 1.630 for interaction, *p* = 0.2069. In **c**, *F* (1, 55) = 10.21 for effect of HA, *p* = 0.0023; *F* (1, 55) = 30.59 for effect of ifenprodil, *p* < 0.0001; and *F* (1, 55) = 0.2520 for interaction, *p* = 0.6177. Values significantly different from control rats are indicated by asterisk and from hyperammonemic rats are indicated by “a”. Bonferroni post-test: ^*^p < 0.05, ^***^p < 0.001, ^aa^*p* < 0.01

We therefore tested whether phosphorylation of PKCζ at Thr560 is altered in hyperammonemic rats. As shown in Fig. 5, phosphorylation of PKCζ at Thr560 was reduced (*p* < 0.05) to 83 ± 6% in control rats and was normalized by treatment with IL-1Ra (Fig. 5a), the Src inhibitor PP2 (Fig. 5b), or by blocking GluN2B with ifenprodil (Fig. 5c).

**Fig. 6:**
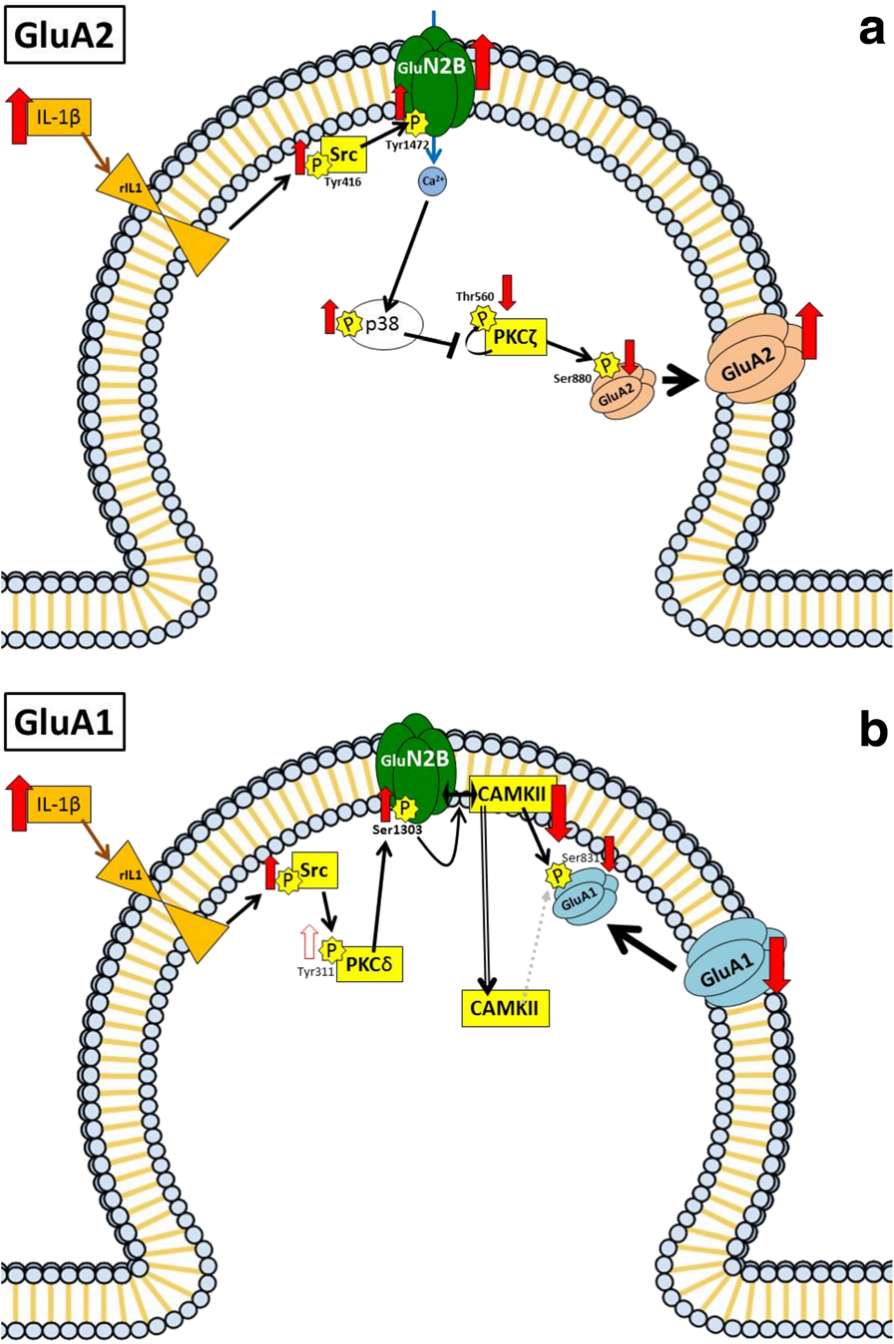
Scheme showing the proposed mechanisms for the IL-1β-induced alterations in membrane expression of GluA2 and GluA1 subunits of AMPA receptor in hippocampus of hyperammonemic rats. Hyperammonemia increases IL-1β, enhancing activation of IL-1 receptor. This leads to activation of Src, reflected in increased phosphorylation of Tyr416. **a** Src in turn enhances phosphorylation at Tyr1472 and membrane expression of GluN2B, which leads to activation of p38. Activated p38 binds to and reduces phosphorylation at Thr560 and activity of PKCζ, thus resulting in reduced phosphorylation at Ser880 and enhanced membrane expression of GluA2. **b** Src also activates PKCδ which enhances phosphorylation of GluN2B at Ser1303, reducing membrane expression of CaMKII and phosphorylation at Ser831 and membrane expression of GluA1

Altogether, the above data allow proposing the pathway shown in Fig. 6a for the mechanism by which hyperammonemia increases membrane expression of the GluA2 subunit of AMPA receptors in hippocampus. Hyperammonemia increases IL-1β, enhancing activation of IL-1 receptor. This leads to activation of Src, reflected in increased phosphorylation of Tyr416. Src in turn enhances phosphorylation of GluN2B at Tyr14721 and membrane expression of GluN2B, which leads to activation of p38. Activated p38 binds to and reduces phosphorylation at Thr560 and activity of PKCζ, thus resulting in reduced phosphorylation at Ser880 and enhanced membrane expression of GluA2.

### CaMKII and PKCδ mediate the effects of IL-1β and Src on GluA1

The above results show that changes in phosphorylation at Ser831 and in membrane expression of GluA1 are also mediated by enhanced activation of IL-1 receptor and of Src, but not by the p38-PKC pathway. As Ser831 may be phosphorylated by PKC or by CaMKII [47, 48], we hypothesized that changes in GluA1 would be mediated by CaMKII.

**Fig. 7:**
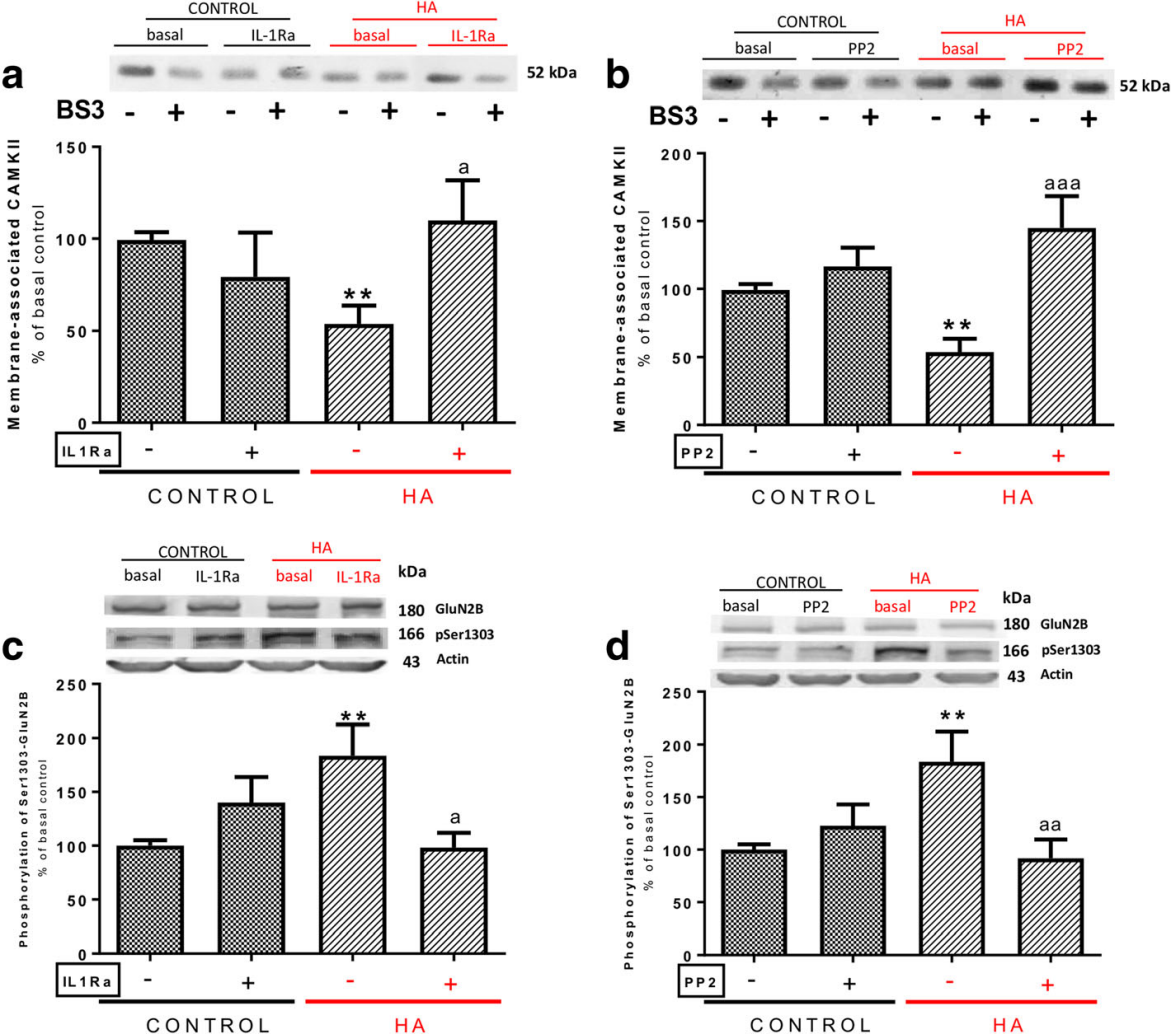
Enhanced activation of IL-1 receptor and Src leads to increased phosphorylation of GluN2B subunit at Ser1303 and reduced membrane-associated CaMKII in hyperammonemic rats. IL-1Ra or PP2 were added to hippocampal slices. CaMKII membrane association (**a**, **b**) and phosphorylation of GluN2B subunit at Ser1303 (**c**, **d**) were analyzed as described in the “Methods” section. Values are expressed as percentage of basal levels in control rats and are the mean ± SEM of 12, 15, 17, and 17 rats per group in **a**, **b**, **c**, and **d** respectively. Data were analyzed by two-way ANOVA. In **a**, *F* (1, 45) = 0.2872 for effect of HA, *p* = 0.5947; *F* (1, 45) = 1.742 for effect of IL-1Ra, *p* = 0.1936; and *F* (1, 45) = 7.909 for interaction, *p* = 0.0073. In **b**, *F* (1, 54) = 0.4278 for effect of HA, *p* = 0.5159; *F* (1, 54) = 18.16 for effect of PP2, *p* < 0.0001; and *F* (1, 54) = 8.633 for interaction, *p* = 0.0048. In **c**, *F* (1, 63) = 1.059 for effect of HA, *p* = 0.3073; *F* (1, 63) = 1.244 for effect of IL-1Ra, *p* = 0.2689; and *F* (1, 63) = 9.532 for interaction, *p* = 0.0030. In **d**, *F* (1, 63) = 1.701 for effect of HA, *p* = 0.1969; *F* (1, 63) = 2.920 for effect of PP2, *p* = 0.0924; and *F* (1, 63) = 7.765 for interaction, *p* = 0.0070. Values significantly different from control rats are indicated by asterisk and from hyperammonemic rats are indicated by “a”. Bonferroni post-test: ^**^p < 0.01, ^a^*p* < 0.05, ^aa^*p* < 0.01, ^aaa^*p* < 0.001

We then assessed if hyperammonemic alters the amount of CaMKII in the membrane. The amount of CaMKII in the membrane is strongly reduced (*p* < 0.01) in membrane of hippocampal slices from hyperammonemic rats, to 54 ± 10% in control rats (Fig. 7). Moreover, this decrease is reversed by treatment with IL-1Ra (Fig. 7a) or by inhibiting Src with PP2 (Fig. 7b).

One of the mechanisms of modulation of membrane expression of CaMKII is phosphorylation of GluN2B at Ser1303. Enhancing this phosphorylation reduces membrane expression of CaMKII [49]. Phosphorylation of GluN2B at Ser1303 is increased in hyperammonemic rats (Fig. 7c, d). Moreover, blocking IL-1 receptor with IL-1Ra (Fig. 7c) or inhibiting Src with PP2 (Fig. 7d) normalizes phosphorylation of GluN2B at Ser1303. This suggests that increased phosphorylation of GluN2B at Ser1303 in hyperammonemic rats leads to reduced membrane expression of CaMKII which, in turn, reduces phosphorylation at Ser831 and membrane expression of GluA1.

Increased phosphorylation of GluN2B at Sr1303 is mediated by IL-1 receptor and by Src. However, Src is a tyrosine kinase, and may not phosphorylate Ser residues. This suggests that a serine-threonine kinase would mediate the effects of Src on phosphorylation of GluN2B at Ser1303 [50]. It has been reported that Src phosphorylates PKCδ at Tyr311, enhancing its activity [51].

**Fig. 8:**
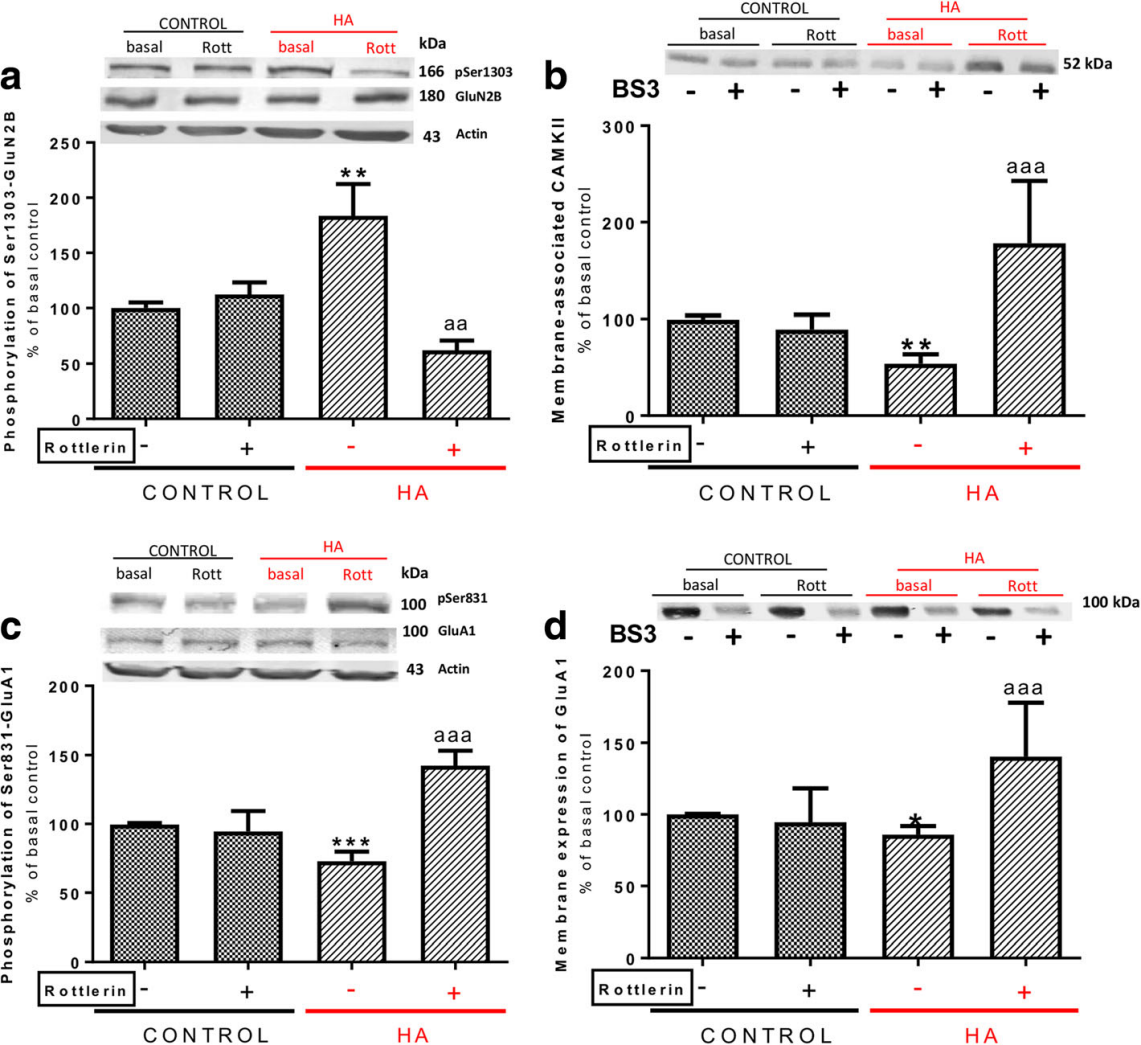
Enhanced activity of PKCδ mediates the increase in GluN2B phosphorylation at Ser1303, the reduced association to membrane of CaMKII, and the reduced phosphorylation at Ser831 and membrane expression of GluA1 in hyperammonemic rats. Rottlerin, an inhibitor for PKCδ, was added to hippocampal slices. Phosphorylation of GluN2B subunit at Ser1303 (**a**) and GluA1 at Ser831 (**c**) and membrane expression of CaMKII (**b**) and GluA1 (**d**) were analyzed as described in the “Methods” section. Values are expressed as percentage of basal levels in control rats and are the mean ± SEM of 15, 10, 29, and 21 rats per group in **a**, **b**, **c**, and **d** respectively. Data were analyzed by two-way ANOVA. In **a**, *F* (1, 55) = 0.5387 for effect of HA, *p* = 0.4661; *F* (1, 55) = 5.649 for effect of rottlerin, *p* = 0.0210; and *F* (1, 55) = 8.403 for interaction, *p* = 0.0054. In **b**, *F* (1, 36) = 1.445 for effect of HA, *p* = 0.2372; *F* (1, 36) = 9.889 for effect of rottlerin, *p* = 0.0033; and *F* (1, 36) = 13.77 for interaction, *p* = 0.0007. In **c**, *F* (1, 113) = 1.353 for effect of HA, *p* = 0.2471; *F* (1, 113) = 11.81 for effect of rottlerin, *p* = 0.0008; and *F* (1, 113) = 16.36 for interaction, *p* < 0.0001. In **d**, *F* (1, 80) = 2.696 for effect of HA, *p* = 0.1045; *F* (1, 80) = 6.059 for effect of rottlerin, *p* = 0.0160; and *F* (1, 80) = 9.431 for interaction, *p* = 0.0029. Values significantly different from control rats are indicated by asterisk and from hyperammonemic rats are indicated by “a”. Bonferroni post-test: ^*^p < 0.05, ^**^p < 0.01, ^***^p < 0.01 ^aa^*p* < 0.01, ^aaa^*p* < 0.001

To assess if increased activity of PKCδ mediates the increase in phosphorylation of GluN2B at Ser1303 in hyperammonemic rats, we analyzed whether a specific inhibitor of PKCδ (rottlerin) reverses this increase. Treatment with rottlerin completely reversed the increase in phosphorylation of GluN2B at Ser1303 in hyperammonemic rats (Fig. 8a). Moreover, rottlerin also increased membrane expression of CaMKII (Fig. 8b) even above (178 ± 75%) in control rats. The increase of CaMKII in the membrane was associated with an increase, also above control rats, of phosphorylation of GluA1 at Ser831 (142 ± 11% in controls, Fig. 8c) and of membrane expression of GluA1 (140 ± 37% in controls, Fig. 8d).

The above data allow proposing the pathway shown in Fig. 6b for the mechanism by which hyperammonemia reduces membrane expression of the GluA1 in hippocampus. Hyperammonemia increases IL-1β, enhancing activation of IL-1 receptor. This leads to activation of Src, reflected in increased phosphorylation of Tyr416. Src in turn activates PKCδ which enhances phosphorylation of GluN2B at Ser1303, reducing membrane expression of CaMKII and phosphorylation at Ser831 and membrane expression of GluA1.

## Discussion

There is increasing evidence that many pathological situations, including neurodegenerative and chronic diseases, lead to neuroinflammation which, in turn, is a main contributor to cognitive impairment in these situations. Neuroinflammation would impair cognitive function by altering neurotransmission. Unveiling the mechanisms by which neuroinflammation alters neurotransmission would allow identifying pathways and therapeutic targets to try to reverse the alterations in neurotransmission and therefore in cognitive function in different pathological situations.

In this study, we have identified two pathways by which neuroinflammation alters membrane expression of the GluA2 and GluA1 subunits of AMPA receptors, respectively, in hippocampus of hyperammonemic rats. It is shown that hyperammonemia increases IL-1β and activation of its receptor, leading to activation of Src which increases phosphorylation at Tyr1472 and membrane expression of GluN2B, which leads to activation of p38. Activated p38 binds to and reduces phosphorylation at Thr560 and activity of PKCζ. This is associated with reduced phosphorylation of GluA2 at Ser880 and enhanced membrane expression of GluA2. On the other hand, activated Src also increases phosphorylation of PKCδ which enhances phosphorylation of GluN2B at Ser1303, reducing membrane expression of CaMKII and phosphorylation at Ser831 and membrane expression of GluA1.

Modulation of the membrane expression of AMPA receptors plays a main role in synaptic plasticity in hippocampus [52]. Long-term potentiation (LTP) is mediated by enhanced and long-term depression (LTD) by reduced membrane expression of AMPA receptors [53, 54]. LTP in hippocampus is considered the bases for spatial learning and memory [55]. Both LTP in hippocampus [56] and spatial learning and memory [7] are impaired in hyperammonemic rats. Altered modulation of GluA1 and GluA2 membrane expression in hyperammonemic rats would be a main contributor to the impairment of LTP and spatial learning.

We show here that the alterations in membrane expression of GluA1 and GluA2 in hyperammonemic rats are a consequence of neuroinflammation and of enhanced activation of IL-1 receptor by increased levels of IL-1β. It has been already shown that high levels of IL-1β impair LTP [57]. Altogether, these data support that in hyperammonemic rats, (and likely in other pathological situations) increased levels of IL-1β in hippocampus alter membrane expression of GluA1 and GluA2 subunits of AMPA receptors, which would lead to impairment of LTP and of spatial learning and memory.

We also identify the intracellular signal transduction pathways by which activation of IL-1 receptor leads to changes in phosphorylation and to opposite effects on membrane expression of GluA1 and GluA2. These pathways are presented in Fig. 6 and in the graphical abstract.

Hyperammonemia increases IL-1β, enhancing activation of IL-1 receptor. This leads to activation of Src, reflected in increased phosphorylation of Tyr416. These steps are common to the pathways leading to altered membrane expression of the GluA1 and GluA2 subunits. The changes in membrane expression of both GluA1 and GluA2 are reversed by blocking the IL-1 receptor with IL-1Ra or by inhibiting Src with PP2, thus confirming the contribution of these steps to the changes in membrane expression.

However, after Src activation, the pathways diverge. The enhanced activity of Src in hyperammonemic rats results in increased phosphorylation of GluN2B at Tyr1472 and membrane expression of GluN2B, which leads to activation of p38. Activated p38 binds to and reduces phosphorylation at Thr560 and activity of PKCζ, thus resulting in reduced phosphorylation at Ser880 and enhanced membrane expression of GluA2 (Fig. 6a). The changes in membrane expression of GluA2 are reversed by blocking the GluN2B-containing NMDA receptors with ifenprodil or inhibiting p38 with SB239063, thus confirming the contribution of these steps to the changes in membrane expression of GluA2 (Fig. 6a). Some reports in the literature support the existence of the steps proposed in Fig. 6a. It has been already shown that Src phosphorylates GluN2B at Tyr1472 and this increases its membrane expression [42] that enhanced membrane expression of GluN2B leads to enhanced phosphorylation and activity of the MAP kinase p38 [43] and that activated p38 binds to PKCζ and this prevents auto-phosphorylation of PKCζ at Thr560, thus reducing its activity [46]. We show here that all these steps are induced sequentially in hyperammonemic rats by activation of IL-1 receptor, leading to increased membrane expression of GluA2.

Moreover, these steps would occur in neurons and not in astrocytes. This is supported by the report of Srinivasan et al. [58], who showed that IL-1β activates the p38 signaling pathway in hippocampal neurons, in contrast to the activation of NF-kB in hippocampal astrocytes, demonstrating cell type-specific signaling responses to IL-1 in the brain and yielding distinct functional responses. However, Srinivasan et al. [58] did not tested if activation of p38 by IL-1β is a direct effect or it is mediated by some previous steps. We show here that increased phosphorylation of p38 is reduced by blocking the IL-1 receptor, by inhibiting Src, or by blocking the NR2B subunit of NMDA receptors, supporting that activation of p38 by IL-1β is mediated by Src and NR2B.

Altered membrane expression of GluA1 is also mediated by activation of IL-1 receptor and Src, but after this step, the pathway is different than for GluA2. Increased activity of Src in hyperammonemic rats also activates PKCδ which enhances phosphorylation of GluN2B at Ser1303, reducing membrane expression of CaMKII and phosphorylation at Ser831 and membrane expression of GluA1. These changes are reversed by blocking the IL-1 receptor with IL-1Ra and by inhibiting Src with PP2 or PKCδ with rottlerin, thus supporting the contribution of the pathway depicted in Fig. 6b in the changes in membrane expression of GluA1. Some reports in the literature support the existence of the steps proposed in Fig. 6b. It has already been reported that Src phosphorylates PKCδ at Tyr311, enhancing its activity [59], and that enhancing phosphorylation GluN2B at Ser1303 reduces membrane expression of CaMKII [49]. We show here that all these steps are induced sequentially in hyperammonemic rats by activation of IL-1 receptor, leading to reduced membrane expression of GluA1.

We show that the steps of the pathway summarized in Fig. 6a are induced sequentially in hyperammonemic rats by activation of IL-1 receptor, leading to increased membrane expression of GluA2. Blocking IL-1β receptor prevents all subsequent steps, indicating that this activation is in the origin of the pathway activation. Inhibiting Src with PP2 prevents changes in phosphorylation and membrane expression of NR2B, in phosphorylation of p38 and of PKCζ and phosphorylation and membrane expression of GluA2, indicating that activation of Src precedes all these steps. Similarly, blocking NR2B with ifenprodil prevents changes in phosphorylation of p38 and of PKCζ and phosphorylation and membrane expression of GluA2, indicating that changes in NR2B precede these steps. Finally, inhibiting p38 with SB239063 prevents changes in phosphorylation and membrane expression of GluA2. Altogether, these results show that the hyperammonemia induces sequential activation of the pathway depicted in Fig. 6a to modulate membrane expression of GluA2. Similarly, hyperammonemia induces sequential activation of the pathway depicted in Fig. 6b to modulate membrane expression of GluA1. Although the activation of these pathways occurs sequentially, it occurs very rapidly. The phosphorylation and membrane expression of GluA1 and GluA2 AMPA receptor subunits is very dynamic and may change rapidly in response to synaptic activity or other stimuli. The data reported here show that, under the conditions used in the present work, hyperammonemia alters these pathways as summarized in Fig. 6.

The above data demonstrate that neuroinflammation in hippocampus induced by hyperammonemia alters glutamatergic neurotransmission by altering membrane expression of AMPA and NMDA receptor subunits. Moreover, this is mediated by increased levels of IL-1β and of activation of IL-1 receptor and we identify the intracellular pathways involved.

It is noteworthy that neuroinflammation may also alter AMPA receptors by other mechanisms. TNF-α also may alter membrane expression of AMPA receptor subunits. Moreover, the effects of IL-1β and of TNF-α on membrane expression of AMPA receptors are the opposite. IL-1β reduces membrane expression of the GluA1 subunit while TNF-α increases GluA1 but reduces GluA2 membrane expression [29, 60–62]. This indicates that different types or grades of neuroinflammation may lead to different alterations in AMPA receptor membrane expression depending on the prevalence of IL-1β or TNF-α effect [29, 60–62].

TNF-α selectively enhances membrane expression of GluA1 in hippocampal neurons and the proportion of GluA2-lacking receptors, resulting in AMPA receptors with different properties becoming calcium-permeable, inwardly rectifying and inhibited by polyamines [61]. In vivo nanoinjection of TNF-α in rats also increases synaptic expression of the GluA1 subunits with a concurrent decrease in the GluA2 subunit [62]. In contrast, IL-1β at high concentrations reduces membrane expression of GluA1 [29]. The results reported here show that, in hyperammonemic rats, increased levels of IL-1β reduces GluA1 and enhances GluA2 membrane expression through activation of IL-1 receptor.

It is also noteworthy that, although the effects of IL-1β and TNF-α on membrane expression of AMPA receptors are the opposite, both increased levels of IL-1β and of TNF-α result in altered glutamatergic neurotransmission by altering AMPA receptor function. Sustained overexpression of either IL-1β or TNF-α impairs hippocampal LTP [63]. Increased levels of IL-1β also impair spatial learning and memory [17, 64]. Some reports suggest that TNF-α also impairs spatial learning [65, 66].

A potential limitation of the present work is that the effects reported could be limited to a certain period of time in the progression of chronic hyperammonemia. The present work has been performed at 4–5 weeks of hyperammonemia. However, the type or intensity of neuroinflammation could be different at longer times of hyperammonemia, triggering other mechanisms. The above reports indicate that neuroinflammation may alter hippocampal neurotransmission and spatial learning by different mechanisms depending on the type and grade of neuroinflammation, for example on the relative contribution of the increases in IL-1β and TNF-α [17, 29, 60–66]. This may explain the discrepancy between the effects induced by 4–5 weeks of hyperammonmeia reported here and those reported in [7] showing increased GluA1 and reduced GluA2 membrane expression at 8 weeks of hyperammonemia. We have preliminary results showing that neuroinflammation in hyperammonemic rats is dynamic and changes with time, resulting in a different pattern of inflammatory markers at different types. Dynamic changes in neuroinflammation have been also reported in situations such as Parkinson’s disease [67], stroke [68, 69], ischemia [70], amyotrophic lateral sclerosis, AIDS, and multiple sclerosis [71]. These dynamic changes in neuroinflammation and its consequences on neurotransmission suggest that the treatments to reverse neuroinflammation-induced cognitive impairment should be different depending on the type and grade of neuroinflammation reached, which would be different in different pathological situations.

We show that in rats with chronic moderate hyperammonemia, similar to that present in patients with liver cirrhosis, neuroinflammation in hippocampus alters membrane expression of AMPA receptors mainly through activation of IL-1 receptor by increased levels of IL-1β. Blocking IL-1 receptor with the endogenous antagonist IL-1Ra reverses completely the alterations in AMPA receptor membrane expression. It has been shown that IL-1Ra also prevents the impairment of LTP by IL-1β [63]. This suggests that blocking this receptor would also restore spatial learning impaired by overexpression of IL-1β in different in pathological situations associated with neuroinflammation, including hyperammonemic rats, and possibly, patients with minimal hepatic encephalopathy.

## Conclusions

In summary, this work identifies two pathways by which neuroinflammation alters glutamatergic neurotransmission in hippocampus. Increased IL-1β and activation of its receptor leads to activation of Src, increased membrane expression of GluN2B, and activation of p38 which reduces activity of PKCζ and phosphorylation of GluA2 at Ser880, increasing membrane expression of GluA2. Activated Src also increases phosphorylation of PKCδ which enhances phosphorylation of GluN2B at Ser1303, reducing membrane expression of CaMKII and phosphorylation at Ser831 and membrane expression of GluA1. These steps could be targets to normalize neurotransmission in hyperammonemia and other pathologies associated with increased IL-1β by acting, for example, on p38 or PKCδ.

## Abbreviations

BS3: Bis (sulfosuccinimidyl) suberate
CAMKII: Calcium-calmodulin dependent protein kinase II
GluA1: Glutamate ionotropic receptor AMPA type subunit 1
GluA2: Glutamate ionotropic receptor AMPA type subunit 2
GluN2B: Glutamate ionotropic receptor NMDA type subunit 2B
IL-1Ra: Interleukin 1 receptor antagonist
LTP: Long-term potentiation
PKCδ: Delta isoform of protein kinase C
PKCζ: Zeta isoform of protein kinase C
PP2: 4-Amino-3-(4-chlorophenyl)-1-(t-butyl)-1H-pyrazolo[3,4-d]pyrimidine
SB239063: Trans-1-(4-hydroxycyclohexyl)-4-(4-fluorophenyl)-5-(2-methoxypyridimidin-4-yl)imidazole
